# Fishing the Targets of Bioactive Compounds from *Psidium guajava* L. Leaves in the Context of Diabetes

**DOI:** 10.3390/ijms24065761

**Published:** 2023-03-17

**Authors:** Elixabet Díaz-de-Cerio, Francisco Girón, Alfonso Pérez-Garrido, Andreia S. P. Pereira, José Antonio Gabaldón-Hernández, Vito Verardo, Antonio Segura Carretero, Horacio Pérez-Sánchez

**Affiliations:** 1 Department of Nutrition and Food Science, University of Granada, Campus of Melilla, 52005 Melilla, Spain; 2 Department of Human Nutrition and Food Technology, Universidad Católica de Murcia UCAM, Campus de los Jerónimos, 30107 Guadalupe, Spain; 3 Structural Bioinformatics and High Performance Computing Research Group (BIO-HPC), Universidad Católica San Antonio de Murcia (UCAM), 30107 Guadalupe, Spain; 4 Department of Biochemistry, Genetics and Microbiology, University of Pretoria, Pretoria 0083, South Africa; 5 Molecular Encapsulation Research Group, Universidad Católica San Antonio de Murcia (UCAM), 30107 Guadalupe, Spain; 6 Department of Nutrition and Food Science, University of Granada, Campus of Cartuja, 18071 Granada, Spain; 7 Institute of Nutrition and Food Technology ‘José Mataix’, Biomedical Research Center, University of Granada, Avda del Conocimiento Sn., 18100 Armilla, Spain; 8 Department of Analytical Chemistry, Faculty of Sciences, University of Granada, Avd. Fuentenueva s/n, 18071 Granada, Spain

**Keywords:** diabetes mellitus, guava, in silico, leaves, phenolic compounds, *Psidium guajava* L.

## Abstract

*Psidium guajava* L. (guava) leaves have demonstrated their in vitro and in vivo effect against diabetes mellitus (DM). However, there is a lack of literature concerning the effect of the individual phenolic compounds present in the leaves in DM disease. The aim of the present work was to identify the individual compounds in Spanish guava leaves and their potential contribution to the observed anti-diabetic effect. Seventy-three phenolic compounds were identified from an 80% ethanol extract of guava leaves by high performance liquid chromatography coupled to electrospray ionization and quadrupole time-of-flight mass spectrometry. The potential anti-diabetic activity of each compound was evaluated with the DIA-DB web server that uses a docking and molecular shape similarity approach. The DIA-DB web server revealed that aldose reductase was the target protein with heterogeneous affinity for compounds naringenin, avicularin, guaijaverin, quercetin, ellagic acid, morin, catechin and guavinoside C. Naringenin exhibited the highest number of interactions with target proteins dipeptidyl peptidase-4, hydroxysteroid 11-beta dehydrogenase 1, aldose reductase and peroxisome proliferator-activated receptor. Compounds catechin, quercetin and naringenin displayed similarities with the known antidiabetic drug tolrestat. In conclusion, the computational workflow showed that guava leaves contain several compounds acting in the DM mechanism by interacting with specific DM protein targets.

## 1. Introduction

Diabetes mellitus (DM) is one of the most serious and increasing health disorders in the world. Today, this disease affects 382 million people, and it is expected that the number of affected could rise to 592 million people by 2035 [1]. The cause of clinical diabetes is due to a deficiency of the effect of insulin at the tissue level and it is usually accompanied by an increase in oxidative stress. This deficiency is caused by an autoimmune destruction or by the dysregulation of insulin release from the pancreatic B-cells (type 1 and 2, respectively) [2]. Therefore, treatment of DM is based on the use of clinical drugs which reduce blood glucose levels.

Furthermore, there is evidence that herbal medicines possess diabetic inhibitory properties through different mechanisms such as α-glucosidase, α-amylase, dipeptidyl peptidase IV (DPP-4), and protein tyrosine phosphatase 1B (PTP-1B) inhibition, as well as the activation of peroxisome proliferator-activated receptor γ (PPARG) [3]. In many plants, these effects have been associated with the presence of bioactive compounds which could be effective as adjuvant in diabetes therapy [4]. Regarding the plant drugs used in traditional medicine, the leaves of *Psidium guajava* L. have been widely employed as hypoglycaemic agents [2].

Guava tree (*P. guajava* L.) is originally from Mexico, although it can grow in tropical and subtropical conditions. Apart from the anti-diabetic effect, different parts of this crop have exhibited in vitro and in vivo properties against several diseases such as diarrhoea and dysentery, and these activities have been related mainly to its phenolic composition, which is greater in the leaves than in the other parts of the tree [5]. Recent studies have revealed the phenolic profile of Spanish guava leaves and the relation of some of the compounds tentatively identified with their anti-diabetic properties [6,7]. Despite the comprehensive study, and the in vitro and in vivo assays conducted, it is still not clear which compounds from the extract are responsible for its anti-diabetic effect. Therefore, we propose in this work to carry out in silico anti-diabetic activity studies to identify the responsible bioactive compounds. We will use two approaches, (a) prediction of the interaction of potentially bioactive molecules with relevant DM targets, providing the characterization of binding modes [8], and (b) prediction of similarity of extract compounds against already known anti-diabetic agents, following the principle *“similar compounds bind to similar targets”* [9]. Based on these premises, the purpose of this work was to evaluate in silico the potential of every phenolic compound present in Spanish guava leaves against the principal targets related to DM.

## 2. Results

### 2.1. Identification of the Phenolic Compositions

Tentative identification of phenolic compounds present in guava leaves via high performance liquid chromatography coupled to electrospray ionization and quadrupole time-of-flight mass spectrometry (HPLC-ESI-QTOF-MS) was accomplished due to a previous work performed by our research group [7] and data are summed up in Table 1. Due to the nature of the phenolic compounds present in the leaf extracts, both negative and positive ionization modes are employed in Table 1 for identification [10]. Despite being detectable by both ionization modes, most phenolic subclasses are detected in negative mode because the sensitivity is better and they mainly produce the ion [M-H]^−^ [10,11]. In contrast, positive mode is used for anthocyanins subclass since [M]^+^ is the predominant ion specie generated due to its structure [11].

Furthermore, the identification of the compounds was achieved according to its retention time, mass spectra and literature. According to Table 1, the retention time, calculated and experimental *m*/*z*, molecular formula, the score, and the error (ppm) are data obtained from HPLC-ESI-QTOF-MS for each compound. Briefly, the MassHunter Workstation Software (version B.06.00 Qualitative Analysis, Agilent Technologies (Santa Clara, CA, USA)) reports the score, which means the feasibility between de mass spectra of the measured compound and the molecular formula that is reporting (in terms of accurate mass, isotope abundance pattern and spacing), and the error (ppm) term, which reveals the difference amongst experimental and calculated mass/charge (*m*/*z*). It is noteworthy that the exact mass of the parent ion is characteristic of each compound as well as its fragmentation pattern.

In agreement with our previous study [6], the phenolic families identified in the guava leaves were flavonols, flavan-3-ols, gallic and ellagic acid derivates, and flavanones. In addition, an anthocyanin was identified (cyanidin-3-O-glucoside). 

### 2.2. In Silico Results and Bibliography Searches

**Figure 1:**
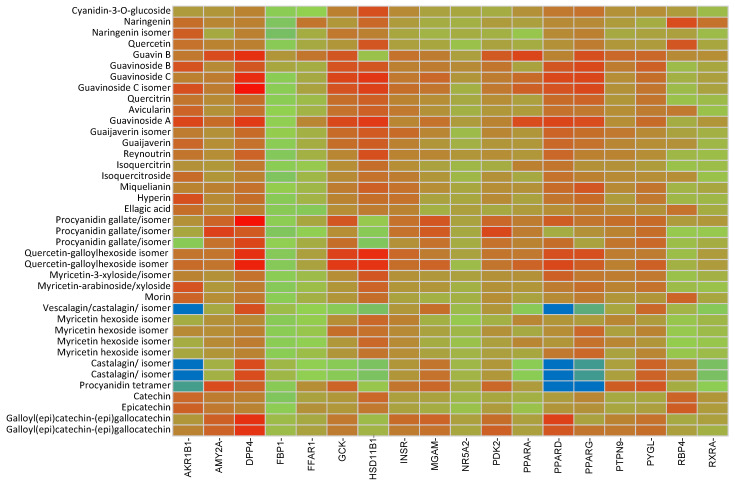
Heat map with the docking results of compounds from guava leaves extract against DM targets. Color scale denotes docking score from blue (no interaction) to red (highest interaction). Each column represents the DM protein target, and each row is assigned to each compound from the extract.

**Figure 2:**
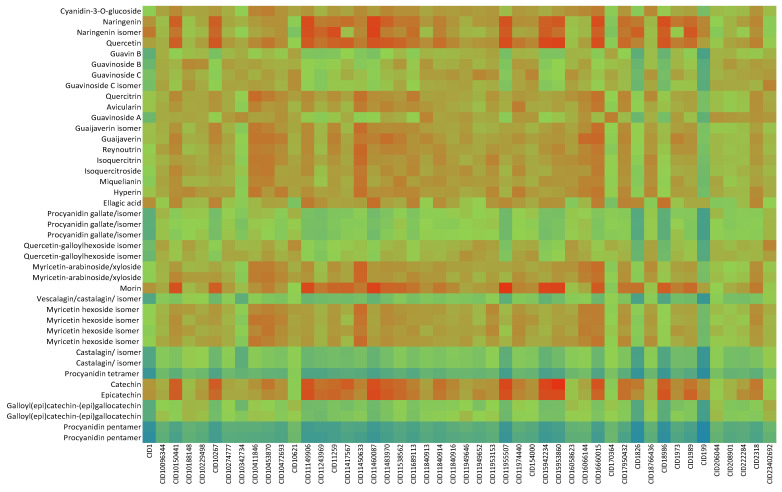
Heat map with the molecular shape similarity results of compounds from *P. guajava* extract against already known anti-diabetic compounds from DIA-DB database. Color scale denotes normalized similarity score from blue (no similarity) to red (highest similarity value). Each column represents each anti-diabetic compound from DIA-DB database (Pubchem ID), while each row is related to each compound from the extract.

Compounds from Table 1 were processed with the DIA-DB server using ligand-similarity-based virtual screening (LBVS) and structure-based virtual screening (SBVS) approaches. The results obtained after docking and molecular shape similarity analyses are shown in Figure 1 and Figure 2, respectively. Based on these results and, to confirm or refute them, a bibliographic review of existing experimental studies for the different compounds present in *P. guajava* leaves was carried out, considering the targets involved in the regulation of glycemia. 

**Figure 3:**
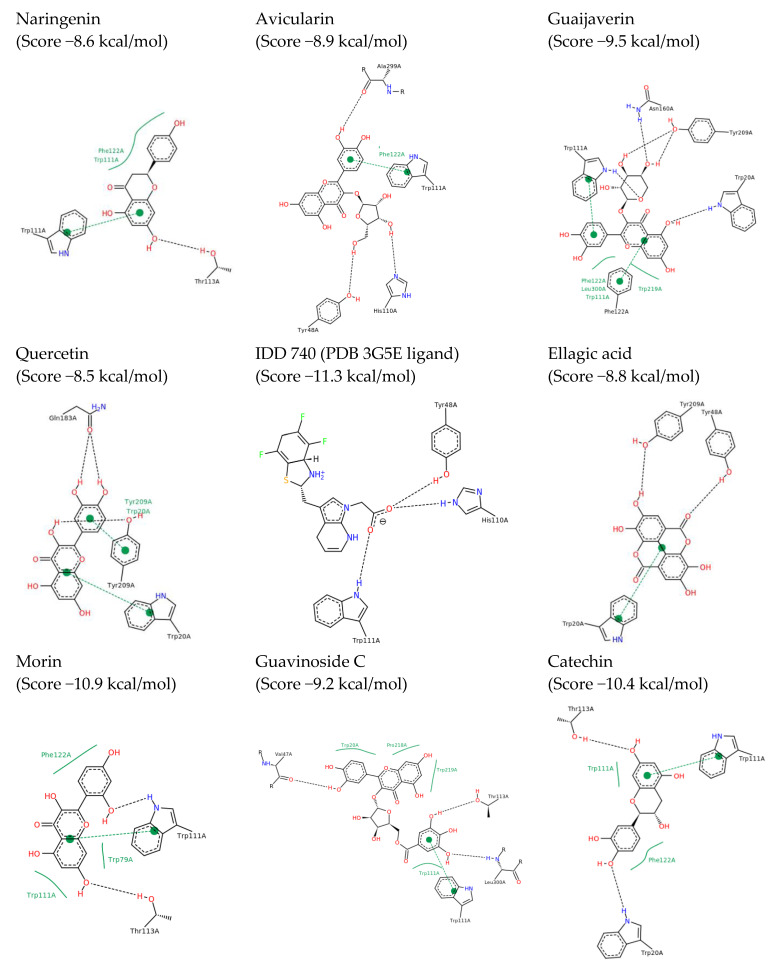
2D representations of docking results for the extract compounds with aldose reductase (PDB 3G5E). Black dashed lines represent hydrogen bonds, green dashed lines aromatic interactions and solid green lines hydrophobic interactions.

The results of the docking analysis showed that aldose reductase (AKR1B1) (protein data bank (PDB) [12]: 3G5E) was the target that presented a more heterogeneous affinity, interacting with: avicularin, (epi)-catechin, ellagic acid, (epi)-gallocatechin isomers 1 and 2, guaijaverin, isoquercitrin, morin, naringenin isomer, quercetin and quercitrin (Figure 3). Compounds morin, naringenin, catechin and quercetin were also observed to have high similarity scores with tolrestat, a known AKR1B1 inhibitor. AKR1B1 is an enzyme of the polyol pathway that has been implicated in diabetic complications. In a study by Anand et al. [13], a *P. guajava* leaf extract was found to inhibit rat lens aldose reductase in vitro.

Likewise, of all the compounds evaluated, naringenin was the one that presented interaction on the highest number of targets: dipeptidyl peptidase-4 (DPP-4) (PDB:4A5S), hydroxysteroid 11-beta dehydrogenase 1 (HSD11B1) (PDB:4K1L), AKR1B1 (PDB:3G5E), and PPARG (PDB:2FVJ) and peroxisome proliferator-activated receptor delta (PPARD) (PDB:3PEQ). There is some evidence in literature supporting the interactions of naringenin with some of these targets identified here. The binding of naringenin to HSD11B1 (PDB:4K1L) stands out with a score value of −8.5 kcal/mol. This result is in line with those obtained in the trials of Ortiz-Andrade et al. [14] in which an IC_50_ of 1000 nM was obtained for this molecule. 

In a recent study by Khan et al. [15], naringenin was found to inhibit aldose reductase in an uncompetitive manner with an IC_50_ of 2.6 µM. Fan et al. [16] demonstrated the ability of naringenin to inhibit DPP4 enzyme in porcine kidney (88% sequence homology with human counterpart) with an IC_50_ of 0.24 µM. Goldwasser et al. [17] showed that naringenin could bind to the ligand–binding domain of PPARG in Hela reporter cell line HG5LN GAL4-PPARG and activate PPARG up to 57% at 80 µM.

**Figure 4:**
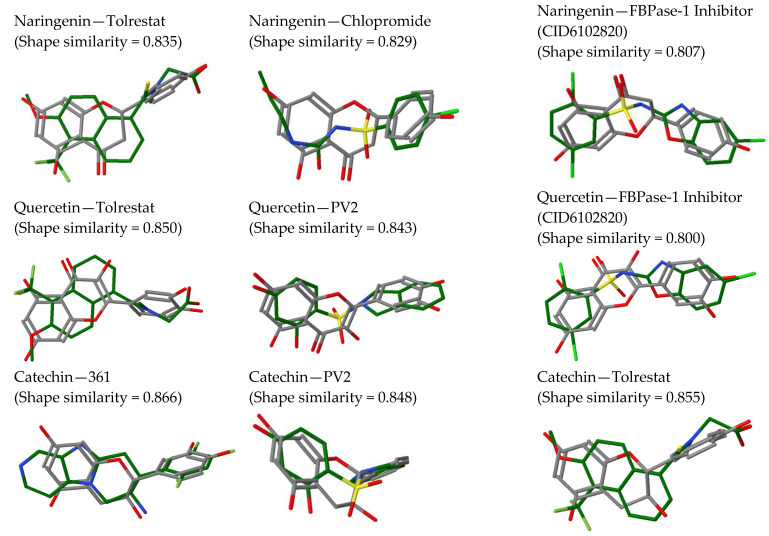
Similarity analyses for the extract compounds naringenin, quercetin and catechin with known/experimental anti-diabetic drugs.

The heterogeneity of interaction shown by naringenin in the docking analysis agrees with the heterogeneity of the analysis of similarity. This indicates that naringenin is similar to other already known anti-diabetic flavonoid compounds such as luteolin or myricetin, as well as to different antidiabetics such as carbutamide and chlorpropamide (sulfonylureas), compound MB07803 (fructose 1,6-bisphosphatase inhibitor), tolrestat (AKR1B1 inhibitor) or picolinate of chromium (III), a drug capable of improving the fluidity of the cell membrane and increasing the rate of internalization of insulin (Figure 4).

The docking analysis showed that quercetin binds to AKR1B1 (PDB:3G5E) with a score value of −8.6 kcal/mol and to HSD11B1 (PDB:4K1L) with a score value of −9.5 kcal/mol, respectively. These results agree with the inhibition tests on AKR1B1 (PDB:3G5E) carried out by Chethan et al. [18], de la Fuente et al. [19] and Ueda et al. [20]. in which quercetin showed IC_50_ values ranging from 14 nM to 248 nM. With regard to HSD11B1 (PDB:4K1L), Torres-Piedra et al. [21] showed that quercetin was able to produce a decrease in the activity of HSD11B1 (PDB:4K1L) of up to 27%.

Several studies indicate that quercetin could also influence protein tyrosine phosphatase (PTP) (PDB:4GE6) and PPARG (PDB:2FVJ) [22,23,24].

This diversity of targets shown by quercetin agrees with the results of similarity analysis in which this compound found high similarity values against other flavonoids (luteolin and myricetin) and with antidiabetics such as the compound PV2 (inhibitor of pyruvate dehydrogenase kinase mitochondrial), the compound MB07803, tolrestat (AKR1B1 inhibitor) or the chromium picolinate (III) (Figure 4).

Quercitrin also showed a good interaction with AKR1B1 (PDB:3G5E) with a score of −8.7 kcal/mol. This interaction was corroborated by the trials of Dhagat et al. [25], Kim et al. [26], Jung et al. [27] and Yoshikawa et al. [28], in which quercitrin gave IC_50_ values between 150 and 340 nM. In addition, the studies carried out by Choi et al. [29] indicate that quercitrin could influence the receptor activated by PPARG (PDB:2FVJ).

Isoquercitrin presented a score value with AKR1B1 (PDB:3G5E) of −8.9 kcal/mol; This value is consistent with the tests developed by Kim et al. [26] in which an IC_50_ value of 320 nM was obtained for this molecule. Also, in their studies with laboratory rats, Brindis et al. [30] showed that isoquercitrin caused a decrease in postprandial glucose peaks similar to that obtained with a dose of acarbose of 5 mg/kg.

Guaijaverin showed a score value of −8.9 kcal/mol in its binding with AKR1B1 (PDB:3G5E). These results agree with laboratory rat tests carried out by Yoshikawa et al. [28], in which they obtained an IC_50_ of 180 nM.

Ellagic acid, on the other hand, was predicted to bind AKR1B1 (PDB:3G5E) and insulin receptor (INSR) (PDB:3EKN) with a score value of −8.8 kcal/mol and −8.2 kcal/mol respectively. In the case of AKR1B1 (PDB:3G5E), trials such as those of Akileshwari et al. [31], Hundsdörfer et al. [32], Naeem et al. [33], Sawant et al. [34] or Kawanishi et al. [35], in which the IC_50_ values of ellagic acid were found between 48 nm and 397 nM again, confirmed the good predictions obtained through the DIA-DB application. Similarly, the INSR inhibition analyses carried out by Sawant et al. [34] where an IC_50_ of 340 nM was obtained further support the predictions made by DIA-DB.

Several studies indicate that ellagic acid could also influence hepatic glycogen phosphorylase (PYGL) (PDB:3DDS) as well as PPARG (PDB:2FVJ) [36,37].

The docking analysis revealed interaction of (epi)-catechin with DPP4, retinol 4 transport protein (RBP4) (PDB:2WR6), AKR1B1 (PDB:3G5E), pancreatic α-amylase (AMY2A) (PDB:4GQR) and HSD11B1 (PDB:4K1L). Of note here is the binding of catechin 1 and 2 to AMY2A (PDB:4GQR) with a score of −8.4 kcal/mol; these results agree with the in vitro studies carried out by Adisakwattana et al. (2011) [38] and Toma et al. (2014) [39] in which they found that catechin reduced the activity of this enzyme by between 5 and 6%. Similarly, catechin and epi-catechin were observed to inhibit the rat lens aldose reductase enzyme in vitro by 38% and 41% at 30 µM, respectively. 

The wide diversity of interactions shown by catechin agrees with the results of similarity analyses, in which this compound showed the highest degree of similarity with other compounds, being very similar to myricetin, luteolin, chromium picolinate (III), and the compounds 361 (DPP4 antagonist), PFT and PV1 (inhibitors of the HSP90 thermal shock protein), PV0, PV2 and PV8 (inhibitors of mitochondrial pyruvate dehydrogenase kinase) and MB07803 (Figure 4).

Geraniin 1 and 2 bind to AMY2A (PDB:4GQR) with score values of −8.9 and −8.2 kcal/mol, respectively. These results agree with the tests carried out by Palanisamy et al. [40], in which they found an IC_50_ value of 970 nM for this compound.

Finally, it is worth mentioning compounds guavinoside C and stachyuranin A. The docking analysis showed that guavinoside C binds with good score values to: DPP4 (PDB:4A5S), intestinal maltase-glucoamylase (MGAM) (PDB:3L4Y), pyruvate dehydrogenase kinase (PDK2) (PDB:4MPC), PTP (PDB: 4GE6), AMY2A (PDB:4GQR), glucokinase (GCK) (PDB:3IMX), HSD11B1 (PDB:4K1L), AKR1B1 (PDB:3G5E) and INSR. In the case of stachyuranin A, the range of unions is smaller but not negligible, and potential targets identified were DPP4 (PDB:4A5S), PYGL (PDB:3DDS), (AMY2A) (PDB:4GQR) and the insulin receptor. 

The analysis of similarity indicated that neither of the two molecules had high similarities with any of the known antidiabetic compounds. In addition, no bibliographic references regarding the antidiabetic activity of these compounds were found. 

## 3. Discussion

Treatment of streptozotocin-/alloxan-induced diabetic rats with *P. guajava* extracts in vivo is associated with a reduction in hyperglycaemia. Several protein targets identified in this study could assist in reducing hyperglycaemia through insulin sensitization and regulation of glucose homeostasis. Compounds naringenin, (epi)-catechin, guavinoside C and stachyuranin A were identified as DPP4 inhibitors. Inhibition of DPP4 would increase the half-life of the incretin hormones and thereby increase insulin secretion, thus allowing time to normalize blood glucose levels [41]. Compounds naringenin, quercetin, (epi)-catechin) and guavinoside C, through their inhibition of 11-beta-HSD1, could inhibit glucose production by the liver and improve glucose-dependent insulin sensitivity [42]. Similarly, in a study by Shen et al. [43], a *P. guajava* extract was found to decrease fructose-1,6-bisphosphatase (FBP1) activity, an enzyme also responsible for glucose production by the liver, and in this study naringenin, quercetin and (epi)-catechin were observed to share a high similarity with MB07803, an FBP1 inhibitor. 

*P. guajava* extracts have been observed to stimulate glucose uptake by hepatocytes, adipocytes, myotubes and intestinal cells possibly through regulation of the insulin signaling pathway [13,43,44,45,46]. PTP1B disrupts the insulin signaling pathway and thus treatment with inhibitors would result in insulin sensitization and improve glucose homeostasis [47]. On the other hand, activation of INSR by agonists will stimulate the insulin signaling pathway, thereby improving insulin sensitivity, and promoting glucose uptake by the tissues [48]. Quercetin and guavinoside C were identified as inhibitors for PTP1B, while ellagic acid and stachyuranin A were found to interact with INSR. Postprandial blood glucose levels may also be decreased through the inhibition of AMY2A and MGAM, two enzymes responsible for carbohydrate digestion. (Epi)-catechin and stachyuranin A were identified as AMY2A inhibitors, while guavinoside C was identified as an inhibitor of both AMY2A and MGAM. In vitro studies by Liu et al. [49], Oghogho and Nimenibo-Udia [50] and Wang et al. [51] with porcine pancreatic alpha-amylase and yeast/rat intestinal alpha-glucosidase showed good inhibitory activity by *Psidium guajava* extracts, comparable to positive control acarbose. 

In addition to reducing hyperglycaemia, *P. guajava* extracts have also been shown to improve the associated hyperlipidaemia. Treatment with *P. guajava* extracts is associated with a reduction in total cholesterol, triglycerides, low-density lipoprotein (LDL) and very-low-density lipoprotein (VLDL) while increasing high-density lipoprotein (HDL) levels in the blood of diabetic rats [52,53,54]. The PPARs play various roles in lipid metabolism by regulating the genes involved in lipogenesis, triglyceride synthesis, reverse cholesterol transport, lipolysis, and fatty acid oxidation. Quercetin, quercitrin and ellagic acid were found to bind PPARG, while naringenin was found to bind both PPARD and PPARG.

In conclusion, DIA-DB web server was used to process information about 73 phenolic compounds present in the extract of guava leaves and to predict their potential bioactivity in the context of DM. After detailed analyses, catechin, quercetin and naringenin showed the highest molecular shape similarity values against already available antidiabetic drugs. In addition, we reported several compounds that act in the DM mechanism through the interaction with specific DM protein targets. Some of them are well known phenolic compounds in guava leaves, such as catechin, ellagic acid, naringenin, guavinoside C, and quercetin and its derivatives guaijaverin and isoquercitrin. However, guaijaverin and isoquercitrin are specific of guava leaves extract, although these were not previously reported in the DM context. In addition, a compound such as stachyuranin A that has not been previously identified in guava leaves, has demonstrated to contribute to the anti-diabetic properties of the leaves. In addition, the bibliographic analysis confirms the validation of the DIA-DB predictions. Finally, this work paves the way for the isolation or selective extraction of some of the specific compounds reported here, and their application as nutraceuticals and/or food additives and for further in vivo studies with target compounds.

## 4. Materials and Methods

### 4.1. Plant Material and Sample Preparation

Middle age intense green leaves were collected in Motril, Spain (36°44′43″ N 3°31′14″ W), in February 2015. The samples were air-dried at room temperature, ground and extracted as follows. Briefly, 0.5 g of sample were extracted with 15 mL of ethanol/water 80/20 (*v*/*v*) via ultrasound-assisted extraction using a sonicator Branson B3510 for 10 min. Then, samples were centrifuged for 15 min at 6000 rpm, the surnatan was collected and the extraction was repeated two times more on the residue. The surnatants were pooled and evaporated, and the residues were re-dissolved in 2 mL of methanol/water 1/1 (*v*/*v*) [6].

### 4.2. HPLC-ESI-QTOF-MS Analyses

Chromatographic analyses were performed using an HPLC Agilent 1260 series (Agilent Technologies, Santa Clara, CA, USA) equipped with a binary pump, an online degasser, an autosampler, and a thermostatically controlled column compartment. Moreover, MS analyses were carried out using a 6540 Agilent Ultra-High-Definition Accurate-Mass Q-TOF-MS coupled to the HPLC, equipped with an Agilent Dual Jet Stream electrospray ionization (Dual AJS ESI) interface. The phenolic compounds were separated using a a Poroshell 120 EC-C18 (4.6 mm × 100 mm, particle size 2.7 μm) (Agilent Technologies).

All the phenolic compounds from *P. guajava* L. leaves were ionized in negative mode, and analyzed using the chromatographic method described by Díaz-de-Cerio et al. (2016) [6]. The gradient elution was carried out using water containing 1% acetic acid as solvent system A and acetonitrile as solvent system B, and applied as follows: 0 min, 0.8% B; 2.5 min, 0.8% B; 5.5 min, 6.8% B; 11 min, 14.4% B; 17 min, 24% B; 22 min, 40% B; 26 min, 100% B, 30 min, 100% B; 32 min, 0.8% B; 34 min, 0.8% B. The sample volume injected was 5 μL and the flow rate used was 0.8 mL min^−1^. The following MS conditions were applied: nebulizer pressure, 50 psi; gas drying temperature, 370 °C; drying gas flow (N2), 12.0 L/min; capillary voltage, 3500 V; fragmentor voltage and scan range were 3500 V and *m*/*z* 50–1500, respectively. Automatic MS/MS experiments were carried out using the followings collision energy values: *m*/*z* 100, 30 eV; *m*/*z* 500, 35 eV; *m*/*z* 1000, 40 eV; and *m*/*z* 1500, 45 eV. However, anthocyanin compounds were ionized in the positive mode using the chromatographic method proposed by Gómez-Caravaca et al. (2013) [55]. 

The phenolic compounds have been identified according to the data previously published [6] and considering their experimental and calculated *m*/*z*, fragments, molecular formula, score and error (ppm).

### 4.3. In Silico Approaches

We used the DIA-DB [56] web server (http://bio-hpc.ucam.edu/dia-db (accessed on 2-December-2021)) to predict the antidiabetic activity of compounds. DIA-DB uses two different approaches, namely LBVS and SBVS.

LBVS methods exploit all existing available information (structure, physicochemical parameters, binding affinities, etc.) about known active and inactive compounds. DIA-DB exploits shape information for checking existence in the database of compounds similar to the ones used in the input query. For that purpose, DIA-DB uses internally the shape complementary tool weighted Gaussin algorithm (WEGA) [57].

SBVS identifies compounds which can bind to a target protein with high affinity. This is achieved by determining the optimal binding position by docking each query molecule to a database of protein targets involved in diabetes and available in DIA-DB and then ranking the compound–targets interactions according to their estimated binding affinity values, namely docking scores. The SBVS protocol implemented in DIA-DB employs the Autodock Vina docking program [58]. Vina finds well-binding ligands for a protein receptor of known structure in an input database that contains the three-dimensional structures of many ligands. Each ligand of the database is docked into the whole surface of the protein using an all-atom representation of the protein and ligand.

### 4.4. Bibliography Searches

Based on the obtained SBVS and LBVS DIA-DB predictions, we checked a posteriori the existence of bibliographical references confirming our predictions. 

## Figures and Tables

**Table 1:** Identification of phenolic compounds in *Psidium guajava* L. leaves by HPLC-DAD-ESI-QTOF-MS.

No.	Compound	rt (min)	*m*/*z* Exp	*m*/*z* Calc	Molecular Formula	Score	Error (ppm)
	Negative mode						
1	Galloyl-Hexahydroxydiphenoyl (HHDP) glucose isomer 1	1.93	481.06	481.34	C_20_H_18_O_14_	96.51	−2.55
2	HHDP glucose isomer 2	2.14	481.06	481.34	C_20_H_18_O_14_	99.09	−0.19
3	HHDP glucose isomer 3	2.52	481.06	481.34	C_20_H_18_O_14_	97.21	−2.24
4	Prodelphinidin B isomer	3.85	609.13	609.51	C_30_H_26_O_14_	97.84	−1.7
5	Gallic acid	4.02	169.01	169.11	C_7_H_6_O_5_	99.27	0.37
6	Pedunculagin/Casuariin isomer 1	5.87	783.07	783.53	C_34_H_24_O_22_	98.57	−1.29
7	Prodelphinidin Dimer isomer 1	7.27	593.13	593.51	C_30_H_26_O_13_	96.51	−2.35
8	(epi)-gallocatechin isomer 1	7.81	305.07	305.26	C_15_H_14_O_7_	95.55	−3.32
9	Vescalagin/castalagin isomer	7.95	933.07	933.62	C_41_H_26_O_26_	99.19	−0.79
10	Prodelphinidin Dimer isomer 2	8.12	593.13	593.51	C_30_H_26_O_13_	96.51	−2.35
11	Uralenneoside	9.39	285.06	285.23	C_12_H_14_O_8_	97.80	−2.69
12	Geraniin isomer 1	9.50	951.08	951.64	C_41_H_28_O_27_	99.56	−0.20
13	Pedunculagin/Casuariin isomer 2	9.54	783.07	783.53	C_34_H_24_O_22_	98.39	−1.36
14	Geraniin isomer 2	9.65	951.08	951.64	C_41_H_28_O_27_	99.56	−0.20
15	Procyanidin B isomer 1	10.02	577.14	577.51	C_30_H_26_O_12_	95.68	−2.55
16	Galloyl(epi)catechin-(epi)gallocatechin	10.35	745.14	745.62	C_37_H_30_O_17_	96.90	−0.62
17	Procyanidin B isomer 2	10.36	577.14	577.51	C_30_H_26_O_13_	99.41	−0.61
18	Tellimagrandin I isomer	10.74	785.09	785.55	C_34_H_26_O_22_	99.13	−0.96
19	Pterocarinin A isomer 1	11.00	1067.12	1067.75	C_46_H_36_O_30_	99.82	−0.11
20	Pterocarinin A isomer 2	11.21	1067.12	1067.75	C_46_H_36_O_30_	98.39	−1.26
21	Stenophyllanin A	11.25	1207.15	1207.89	C_56_H_40_O_31_	98.64	−1.08
22	Procyanidin trimer isomer 1	11.25	865.20	865.77	C_45_H_38_O_18_	97.53	−1.59
23	(epi)-catechin	11.26	289.07	289.26	C_15_H_14_O_6_	96.76	−3.18
24	Procyanidin tetramer	11.34	1153.26	1153.03	C_60_H_50_O_24_	99.60	−0.50
25	Procyanidin trimer isomer 2	11.41	865.20	865.77	C_45_H_38_O_18_	97.53	−1.59
26	Guavin A	11.50	1223.14	1223.89	C_56_H_40_O_32_	99.05	0.85
27	Casuarinin/Casuarictin isomer	11.90	935.08	935.64	C_41_H_28_O_26_	97.67	−1.43
28	Galloyl(epi)catechin-(epi)gallocatechin	12.10	745.14	745.62	C_37_H_30_O_17_	96.90	−0.62
29	Procyanidin pentamer	12.14	1441.32	1441.27	C_75_H_62_O_30_	95.66	1.97
30	Galloyl-(epi)catechin trimer isomer 1	12.17	1017.21	1017.87	C_52_H_42_O_22_	99.72	−0.01
31	(epi)-gallocatechin isomer 2	12.33	305.07	305.26	C_15_H_14_O_7_	95.55	−3.32
32	Tellimagrandin I isomer	12.50	785.09	785.55	C_34_H_26_O_22_	98.44	−1.38
33	Vescalagin	12.76	933.07	933.62	C_41_H_26_O_26_	96.33	−0.80
34	Stenophyllanin A isomer	12.93	1207.15	1207.89	C_56_H_40_O_31_	98.37	0.89
35	Galloyl-(epi)catechin trimer isomer 2	12.99	1017.21	1017.87	C_52_H_42_O_22_	98.17	−1.35
36	Myricetin hexoside isomer 1	13.28	479.08	479.37	C_21_H_20_O_13_	98.36	−0.92
37	Stachyuranin A	13.41	1225.16	1225.91	C_56_H_42_O_32_	95.54	1.35
38	Procyanidin gallate isomer	13.52	729.15	729.62	C_37_H_30_O_16_	96.89	−1.91
39	Myricetin hexoside isomer 2	13.68	479.08	479.37	C_21_H_20_O_13_	97.89	−0.08
40	Vescalagin/castalagin isomer	13.84	933.07	933.62	C_41_H_26_O_26_	88.32	−1.57
41	Myricetin-arabinoside/xylopyranoside isomer 1	13.99	449.07	449.34	C_20_H_18_O_12_	98.39	−1.65
42	Myricetin-arabinoside/xylopyranoside isomer 2	14.21	449.07	449.34	C_20_H_18_O_12_	98.02	−1.65
43	Procyanidin gallate isomer	14.56	729.64	577.51	C_30_H_26_O_12_	98.17	−1.73
44	Myricetin-arabinoside/xylopyranoside isomer 3	14.99	449.07	449.34	C_20_H_18_O_12_	98.66	−1.65
45	Myricetin hexoside isomer 3	15.03	479.08	479.37	C_21_H_20_O_13_	97.08	−1.92
46	Myricetin hexoside isomer 4	15.22	479.08	479.37	C_21_H_20_O_13_	97.08	−1.92
47	Myricetin-arabinoside/xylopyranoside Isomer 4	15.60	449.07	449.34	C_20_H_18_O_12_	98.39	−1.65
48	Quercetin-galloylhexoside isomer	15.63	615.10	615.47	C_28_H_24_O_16_	99.16	−0.98
49	Ellagic acid deoxyhexoside	15.84	447.06	447.33	C_20_H_16_O_12_	91.25	−3.19
50	Quercetin-galloylhexoside isomer	16.04	615.10	615.47	C_28_H_24_O_16_	99.16	−0.98
51	Myricetin-arabinoside/xylopyranoside isomer 5	16.19	449.07	449.34	C_20_H_18_O_12_	98.39	−1.65
52	Morin	16.28	301.04	301.23	C_15_H_10_O_7_	97.46	−2.50
53	Myricetin-arabinoside/xylopyranoside isomer 6	16.46	449.07	449.34	C_20_H_18_O_12_	98.39	−1.65
54	Ellagic acid	16.51	301.00	301.19	C_14_H_6_O_8_	98.88	−1.71
55	Hyperin	16.62	463.09	463.37	C_21_H_20_O_12_	96.41	−2.65
56	Quercetin glucoronide	16.72	477.07	477.35	C_21_H_18_O_13_	98.10	−1.83
57	Isoquercitrin	16.95	463.09	463.37	C_21_H_20_O_12_	97.04	−2.33
58	Procyanidin gallate isomer	17.04	729.15	729.62	C_37_H_30_O_16_	96.89	−1.91
59	Reynoutrin	17.50	433.08	433.34	C_20_H_18_O_11_	95.94	−2.90
60	Guajaverin	17.80	433.08	433.34	C_20_H18O_11_	97.99	−1.91
61	Guavinoside A isomer 1	17.96	543.12	544.46	C_26_H_24_O_13_	98.10	−1.77
62	Avicularin	18.21	433.08	433.34	C_20_H_18_O_11_	96.70	−2.20
63	Quercitrin	19.19	447.10	447.37	C_21_H_20_O_11_	95.23	−3.02
64	Myrciaphenone B	19.21	481.10	481.38	C_21_H_22_O_13_	97.20	−2.23
65	Guavinoside C	19.77	585.09	585.45	C_27_H_22_O_15_	97.19	−1.92
66	Guavinoside B isomer 1	20.77	571.15	571.51	C_28_H_28_O_13_	97.26	−2.05
67	Guavinoside A isomer 2	20.70	543.12	543.45	C_26_H_24_O_13_	98.10	−1.77
68	Guavinoside B isomer 2	21.67	571.15	571.51	C_28_H_28_O_13_	97.26	−2.05
69	2,6-dihydroxy-3-methyl-4-O-(6″-O-galloyl-β-D-glucopyranosyl)-benzophenone	21.97	557.13	557.48	C_27_H_26_O_13_	96.93	−2.12
70	Guavin B	22.24	693.11	693.54	C_33_H_26_O_17_	97.82	−1.67
71	Quercetin	22.31	301.04	301.23	C_15_H_10_O_7_	98.90	−1.34
72	Naringenin isomer	26.74	271.06	271.25	C_15_H_12_O_5_	96.09	−3.67
	Positive mode						
73	Cyanidin-3-O-glucoside	3.66	449.11	449.39	C_21_H_21_O_11_	96.97	−2.34

## Data Availability

Not applicable.
